# Community-based approach for a flood preparedness plan in Malaysia

**DOI:** 10.4102/jamba.v11i1.598

**Published:** 2019-02-25

**Authors:** Nur N.M. Shariff, Zety S. Hamidi

**Affiliations:** 1 Academy of Contemporary Islamic Studies, Universiti Teknologi MARA, Malaysia; 2 Institute of Science, Universiti Teknologi MARA, Malaysia

## Abstract

Floods have caused inevitable major disasters around the world as well as in Malaysia. This paper demonstrates that lessons can be taken from the previous flood disasters when developing an effective flood preparedness plan. As a common practice, disaster management is based on a top–down approach or is government-centred. This article attempts to highlight the significance of developing a flood preparedness plan by involving the communities affected. Qualitative analysis was adopted in order to gain in-depth insight of the communities. Two flood-prone communities were chosen: (1) Machang, Kelantan; and (2) Kuala Lipis, Pahang. There were two important things executed by the community for the preparation: (1) community-based disaster risk management; and (2) intensive mutual assistance.

## Introduction

Disaster as defined by United Nations International Strategy for Disaster Reduction (UNISDR 2009) is:

a serious disruption of the functioning of a community or a society involving widespread human, material, economic or environmental losses and impacts, which exceeds the ability of the affected community or society to cope using its own resources. (p. 9)

Disaster can be classified into two types: (1) natural disaster and (2) man-made disaster (Ramli, Mokhtar & Aziz 2014). Regardless of its classification, a community-based approach preparedness plan is applicable for both types.

Starting from the 1st World Conference on Natural Disaster in Yokohama (1994) then followed by Hyogo Framework for Action in 2005, then succeeded by Sendai Framework in 2015, we can witness initiatives or priorities changing from emergency management to disaster risk management (DRM) (Salajegheh & Pirmoradi 2013). From the above three international blueprints on DRM, preparedness is clearly stated as one of its principles (International Decade for Natural Disaster Reduction 1994), its strategic goals (International Strategy for Disaster Reduction 2005) and its priorities for action (United Nations 2015).

**FIGURE 1:**
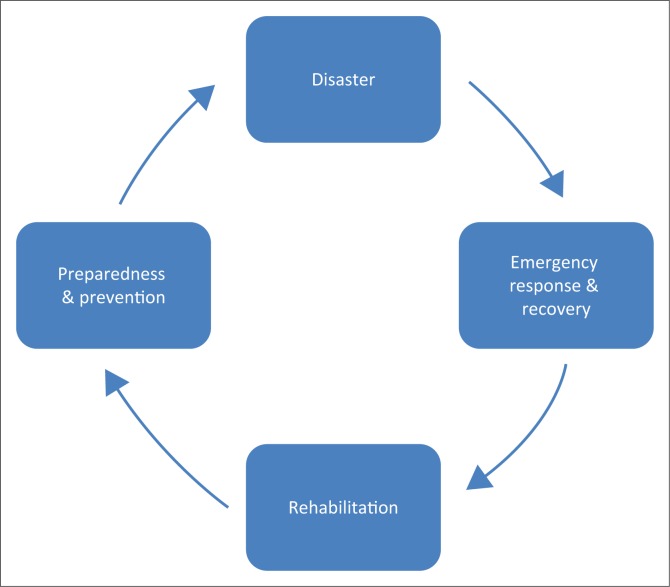
Cycle of disaster.

According to Schipper and Pelling (2006), preparedness is a part of systematic incorporation of DRM, which includes prevention and mitigation. It can be said that preparedness is a state of readiness. Three elements must be presented in the state of readiness, that is, (1) prepare, (2) plan and (3) stay informed. Preparedness is the range of deliberate, critical tasks and activities necessary to build and sustain. The most forgotten process is preparedness and prevention of the disaster (Asanobu 2016; Chan 2012). This can create a cycle of disaster (Figure 1).

The immediate emergency response and recovery process will take part in a case of a disaster. When the situation settles, structured rehabilitations are then employed (the initiatives normally coming from the government, and at the same time, there is a need to improve the operational capability to prevent, protect against, respond to and recover from domestic incidents involving efforts at all levels of government as well as between government and private sectors and non-governmental organisations (NGOs). In the case of Malaysia, there are five phases: (1) prediction, (2) warning, (3) emergency relief, (4) rehabilitation and (5) reconstruction (Othman et al. 2014; Wahab 2012).

In Malaysia, DRM is based on a top–down approach or is government-centred (Chan 2012). This explains why the preparedness plan in Malaysia is reactive rather than proactive (Shariff & Hamidi 2016b). Salleh and Shahran (2016) have stated that one of the biggest challenges for flood disaster management in Malaysia is its heavy dependence on the government machinery. The National Security Division (NSD) as a unit under the Prime Minister’s department is responsible for disaster management. After several disasters (be it natural or man-made disaster), an Executive Order from the Prime Minister was established on 11 May 1997 in which the NSD was bound. The Executive Order is also known as the National Security Council Directive No. 20 on ‘Policy and Mechanism on National Disaster and Relief Management’. The objectives of the Directive are (1) to outline policy on disaster and relief management based on the level of complexity of disaster and (2) to identify the roles and responsibilities of various agencies in establishing management mechanism (Majlis Keselamatan Negara 2016), for instance, the Public Works Department, Welfare Department, Statistics Department, Drainage and Irrigation Department, Malaysian Medical Relief Society (MERCY Disaster Risk Reduction Workshop 2016) and many other NGOs.

Starting from 2015, the agency responsible for disaster management has changed from the NSD to the National Disaster Management Agency which is in line with Sendai Framework. It is important to note that there are several committees set at different levels such as federal, state, district and village levels (which is managed by district committees). According to Obeta (2014), there are gaps in the top–down approach, such as inadequate sustainable flood control strategies, limited resources for threatened communities, et cetera., just to name a few.

This paper demonstrates that a bottom–up approach or community-based preparedness plan is an effective approach for flood management. By engaging the public and giving the public a more active role, their ability to respond to floods or other disasters effectively and appropriately could be enhanced. In conducting a community-based preparedness plan, the act of volunteerism is a must as an expression of the individual’s involvement in their community (United Nations Volunteers 2011).

## Methodology

The aim of this study is to highlight the significance of developing a flood preparedness plan by involving the community. In this study, qualitative analysis was adopted in order to gain in-depth insight of the communities. In order to obtain a reliable data analysis, the triangulation technique was applied. The assessment was done for a duration of 6–10 months of preparation before the expected flood in December 2016 by interviewing around 50 community members (interviews which includes key informants by applying snowballing technique). For instance, semi-structured interview on their pre-, during and post- experience; observation of their preparation in the village; course participation with MERCY; field notes; documents and audio-visual materials were used to uncover barriers and benefits (Creswell 2009; McKenzie-Mohr 2008; Williams 2004).

### Brief background of Malaysia

Malaysia is vulnerable to natural disaster threats such as floods, earthquakes, haze, storms and landslides. According to the International Disaster Database report from 1990 to 2014, 62.5% of the most frequent disaster cases happened in Malaysia. Therefore, flood hazard in Malaysia contributes to 98.7% of annual average loss (AAL) compared to other hazards. Annual average loss includes property and crop damage, number of casualties, disease epidemics and other intangible losses (Chan et al. 2002). Consequently, Malaysia has been listed amongst the top ten countries for the number of victims of natural disasters together with Brazil, Burkina Faso, Sri Lanka and Serbia (Guha-Sapir, Hoyois & Below 2014). Despite everything, Department of Irrigation and Drainage has developed flood early warning system through integration of atmospheric, radar hydrology and satellite to provide longer lead-time estimation – ‘Integrated Atmospheric and Radar Satellite Model-based Rainfall and Flood Forecasting (AMRFF)’ (Wahab 2012).

According to an official statement made by His Excellency Deputy Prime Minister at the 3rd United Nations World Conference on Disaster Risk Reduction, end 2014, floods were affecting more than 500 000 people and public infrastructure damages were estimated at $710 000.00. It was the worst ever in the country’s history because the flood water rose to an unprecedented level and areas that have never experienced floods before were also inundated (Yassin 2015). Therefore, two flood-prone communities were chosen: (1) Machang, Kelantan; and (2) Kuala Lipis, Pahang. Machang was chosen because of the number of flood disaster cases. Meanwhile, Kuala Lipis was chosen as it experienced the worst flood disaster in 2014 after the last flood occurrence in 1971, 43 years ago. Kuala Lipis was chosen as it was unexpectedly hit by floods during the end of January 2017.

## Results and discussion

In this paper, two important things were executed by the community for the preparation: (1) community-based disaster risk management (CBDRM); and (2) intensive mutual assistance which was done as early as 6 months before the expected flood occurred (in December 2016).

### Community-based disaster risk management

A number of awareness programmes on floods have been carried out by strategic agencies, that is, Department of Meteorology Malaysia, Department of Public Works and Southeast Asia Disaster Prevention Research Institute, National University of Malaysia (SEADPRI-UKM). Other than that, in order for public to respond accordingly to disasters, safety guidelines have been distributed to schools and community leaders (Wahab 2012).

Because the National Platform on Disaster Management involves the government, NGOs, community-based organisations (CBOs), private sectors and education institutions, their establishment is to drive more comprehensive and proactive multi-hazard approach in identifying, preventing, mitigating and preparing for disaster risks (Wahab 2012).

Community-based disaster risk management is seen as a contribution of an NGO that is a part of Building Resilient Community module by MERCY Malaysia. It is often paired with School Preparedness Programme focusing on school children for disaster awareness (Shariff & Hamidi 2016a). This kind of training is conducted 10 months before the expected flood. The aims of CBDRM are (1) to develop disaster-resilient communities that are elastic and flexible and (2) to promote disaster-resistant communities that are able to prevent hazards from becoming disaster at a certain point (Salajegheh & Pirmoradi 2013).

In conducting the CBDRM, the communities need to understand the flood risk, have the knowledge and technology to mitigate the risk, share flood hazard information and exchange information with other vulnerable communities (Levy & Gopalakrishnan 2007). Training is essential because it exposes the community to several related topics such as climate change, natural elements and disaster, early warning disaster, what-to-do, grab bag for individual and family and health and hygiene. The above-mentioned topics look very common but people tend to overlook them during emergency. Below are the activities for indoor and outdoor settings.

#### Identification of key actors in providing assistance in the disaster – Indoor activity

**FIGURE 2:**
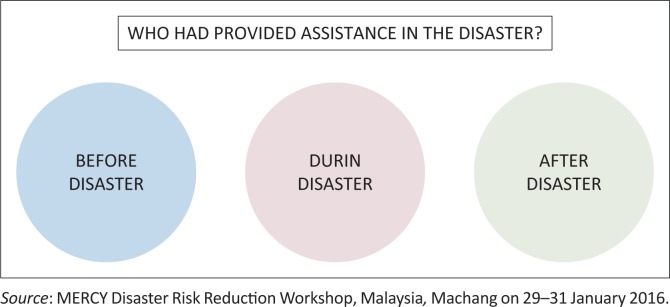
Identification of key actors in providing assistance in the disaster – Separate situations.

Firstly, they were asked to identify who had provided assistance in the disaster with separate situations of ‘before disaster’, ‘during disaster’ and ‘after disaster’ (Figure 2). The community then was divided into several groups. Time was given for the group discussions that was between 15 min and 20 min. The names identified for the situations are mostly government’s instruments – Fire Department, Royal Malaysia Police, Land office, Civil Defence Department, the People’s Volunteer Corps (RELA), Department of Social Welfare, Ministry of Health, Ministry of Communication, social media, political parties, Ministry of Education, private sector, NGOs, villagers, Head of Village, Malaysian Meteorological Department, Department of Registration, MERCY Malaysia and students from various institutions.

**FIGURE 3:**
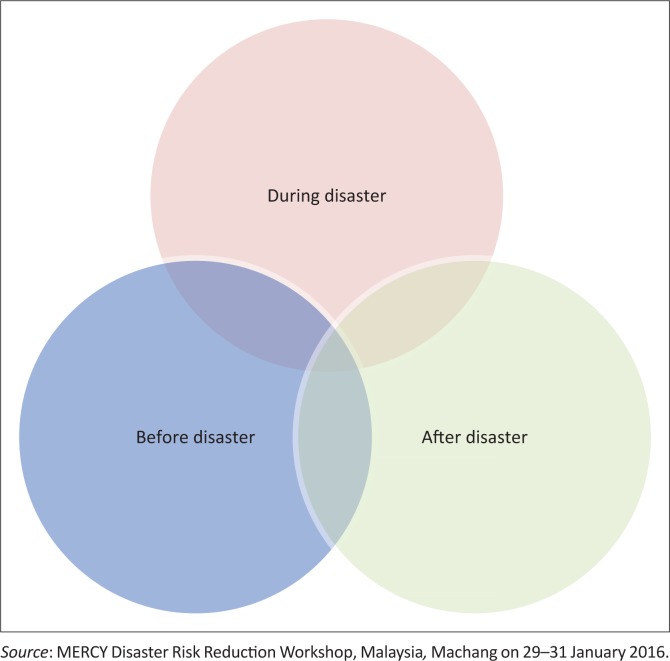
Identification of key actors in providing assistance in the disaster – Overlapped situations.

The last third of the allocated time, the participants were asked to overlap the situations in order to see who provided assistance the most in these three situations (Figure 3). However, one major oversight that most of the groups made was, they did not realise that they themselves should be in the middle of the overlapped circles as key actors. By pointing this out, this activity helped them to envisage that (1) they are as important as others who assisted in the disaster; (2) take charge in the disaster; (3) prepare themselves with relevant knowledge on disaster and (4) plan to equip the community or village with appropriate paraphernalia.

#### Knowing your village – Outdoor activity

After becoming aware of their roles, the participants were directed to another activity which required them to learn about the condition of their villages well. This was done through (1) surveying, (2) mapping, (3) discussion and presentation and (4) planning. Hiwasaki et al. (2014) believed that the integration of indigenous and local knowledge with science is beneficial in order to assist education, policies and actions related to DRM. This practice includes observation, documentation, validation and classification of local and indigenous knowledge. The following are some of the questions identified in this process:

- What are the advantages of your village?
- What are the disadvantages in your village?
- Where is the safest place in your village?
- Where is the most dangerous place in your village?
- What do we remember about the experience of the previous flood?
- How can we reduce the risk of flooding?
- Is there a possibility of flooding?

**FIGURE 4:**
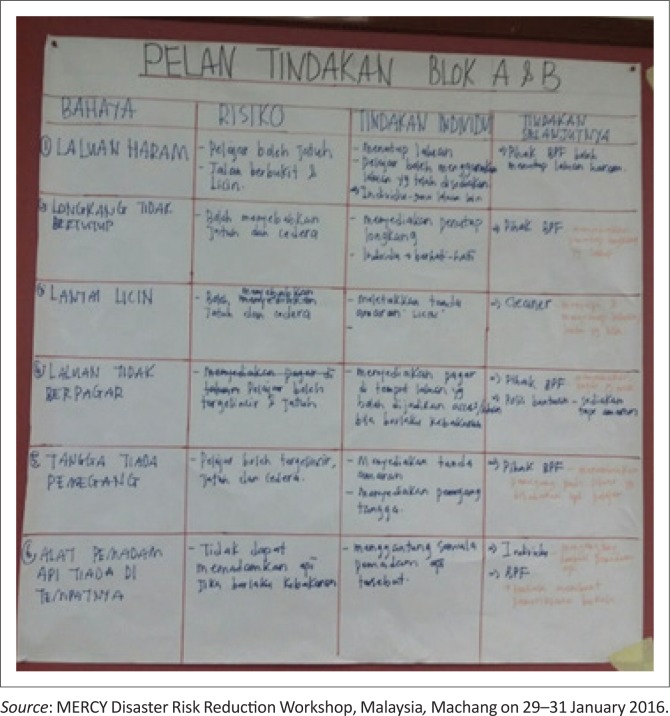
Action plan.

The first step that they needed to take was to get the map of the area or village. Then the community was divided into groups according to the zones. Within the groups, they were assigned specific tasks, for instance, group leader, navigator, photographer, secretary and draftsman. Secondly, they were required to conduct a survey of the area or village. They were asked to identify the environment by sketching a map of the area or village, and at the same time they needed to take photos. Next, they were asked to indicate the danger areas and the advantage and disadvantage areas and finally come up with an action plan. The action plan tabulated information labelled as ‘danger’, ‘risk’, ‘individual action’ and ‘further action’ – this action plan can be altered accordingly (Figure 4).

Finally, the community participated in simulation exercises in order to remind them that flood mitigation strategies and recovery plans should consider community needs (self-identified) through meaningful consultation and participation. The University and MERCY are just facilitators and not problem solvers. There are three types of knowledge ingrained in the community, which are: (1) local wisdom, (2) vulnerability and (3) known or unknown capabilities. Other than exposing them to knowledge on related topics, experience-sharing sessions were also implemented in the training, be it their personal experience as flood survivors or volunteers. This helps the participants to learn from each other’s experiences. This enables them to take the appropriate action as they learnt about equipment and resources (financial and human resources).

### Intensive mutual assistance

It was found that at community level, intensive mutual assistance was taken seriously, which was done as early as six months before the expected flood (for this case, around December 2016). The frequency of this initiative increased towards November as the rainfall was more, for instance, clearing the bushes around the village, clearing any blockage in the small river, clearing and cleaning around public space and between houses. By engaging the public and giving the public a more active role, their ability to respond to floods or other disasters effectively and appropriately could be enhanced.

**FIGURE 5:**
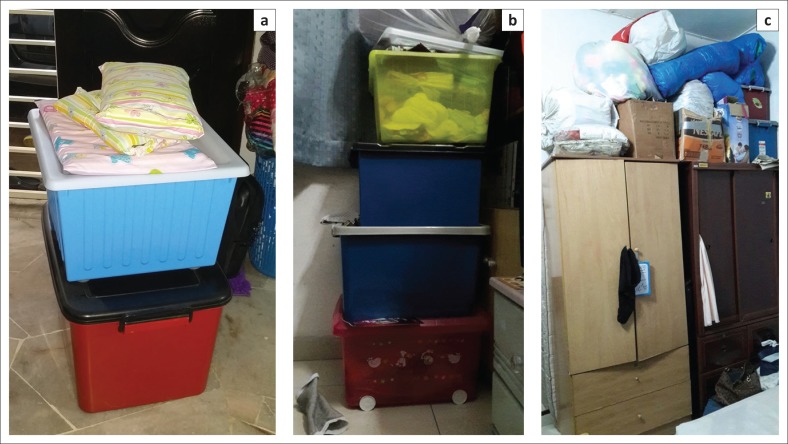
Normal practice in Malaysia. Optimising the space by (a and b) stacking and (c) putting important belongings in plastic containers either in the house or to keep at a neighbour’s house (subject to available space and permission of owner of the house) for 2–3 months.

At household level, 2–3 months before the expected flood, most of the community started to pack their occasional belongings either to keep them in higher places in their houses or to keep them at neighbour’s houses, which are located on higher ground (Figure 5). One month before the expected flood, belongings such as carpets, rugs or anything at floor level should be kept in higher places.

**FIGURE 6:**
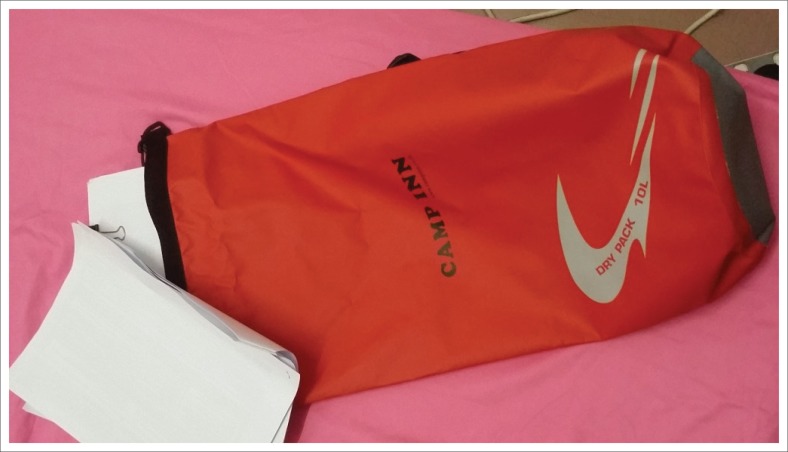
Keeping important documents or even electronic gadgets in a waterproof bag – This bag can serve as a grab bag as well.

Belongings that need to be kept close to the owners, such as important documents, are kept in waterproof bag (Figure 6). Waterproof bags serve as emergency kits containing non-perishable food, torchlight, hygiene kit, stash money, emergency kit, clothes, whistle and extra batteries.

In carrying out such an initiative, participation, trust, solidarity and reciprocity, grounded in a shared understanding and a sense of common obligations are mutually reinforcing values at the heart of governance and good citizenship. Therefore, it is necessary to have the processes of *machizukuri* or community planning that comprises of public consultation and public involvement. *Machizukuri* is a Japanese word where *machi* is a noun, meaning community or neighbourhood. *Tsukuru* is a verb, which means to build. Consequently, *machizukuri* is sometimes defined as a process of community building (Bosman 2007; Issarathumnoon 2004). After all, a community-based approach preparedness plan involves volunteerism that includes informal and formal ways. The key points in volunteering identified are: (1) do small things first, (2) contribute in your own way and (3) carry out sincere connection. Through our observation, we find it important to emphasise coordinated initiatives to avoid waste of time, energy and money.

## Conclusion

This study concluded that a community-based approach for flood preparedness plan depends on: (1) flood frequency; (2) flood severity; (3) community awareness regarding flood hazard – knowledge and experience – that turn into willingness or volunteerism; and (4) types of social unit – preparedness plan for the whole village, which might be slightly different from that of a family or an individual. Flood preparedness plan must meet specific objectives, which are: (1) completing not competing, (2) bridging the gap not redundancy and (3) a holistic approach not ad hoc basis.

Moreover, the preparedness plan must have met basic guideline requirement and must be in accordance with the standard operating procedures (SOP) and emergency response plan (ERP) (Department of Irrigation and Drainage Malaysia 2016; Ramli et al. 2014; Shariff & Hamidi 2016b). The most important lessons are (1) to identify the key actors in providing assistance in the disaster and (2) to learn the advantages and disadvantages of the villages by mapping them. Reiterating by Hiwasaki et al. (2014), the integration of indigenous and local knowledge with science will help to develop resilient communities. In other words, the communities themselves have become the first respondent to the disaster. Having high technology is nothing when the humans are not knowledgable enough to save themselves. Therefore, building a resilient community means that the community has knowledge (either through learning or experience and traditional and local wisdom) about their own vulnerability and capability.
